# Differences in the Analgesic Effect of Opioids on Pain in Cancer Patients With Spinal Metastases

**DOI:** 10.1089/pmr.2023.0018

**Published:** 2023-08-09

**Authors:** Miho Takemura, Kazuyuki Niki, Yoshiaki Okamoto, Hiroshi Tamura, Tomohiro Kawamura, Makie Kohno, Yoshinobu Matsuda, Kenji Ikeda

**Affiliations:** ^1^ Department of Clinical Pharmacy Research and Education, Osaka University Graduate School of Pharmaceutical Sciences, Suita, Japan.; ^2^ Department of Pharmacy, Ashiya Municipal Hospital, Ashiya, Japan.; ^3^ Department of Rehabilitation, and Ashiya Municipal Hospital, Ashiya, Japan.; ^4^ Department of Palliative Care, Ashiya Municipal Hospital, Ashiya, Japan.

**Keywords:** bone metastasis pain, cancer pain management, methadone, opioids, spinal metastasis, tapentadol

## Abstract

**Background:**

Spinal metastasis pain includes both inflammatory and neuropathic pain, and opioids, which have only a μ-opioid receptor-stimulating effect, are generally less effective in neuropathic pain. However, no previous study has been conducted for the comparisons of the efficacy of opioids in treating spinal metastasis pain.

**Objective:**

To compare the efficacy of tapentadol and methadone with other opioids for back pain caused by a metastatic spinal tumor.

**Design:**

Retrospective cohort study.

**Setting/Subjects:**

A total of 274 patients were enrolled, who started a tapentadol extended-release tablet, methadone tablet, hydromorphone extended-release tablet, oxycodone extended-release tablet, or transdermal fentanyl patch for cancer pain due to spinal metastasis in Japan from January 1, 2013 to October 31, 2021.

**Measurements:**

The primary endpoint, the difference in the numerical rating scale (NRS) scores before and seven days after each opioid administration, was compared among the five groups.

**Results:**

In patients with numbness, a decrease of the NRS score on day seven compared with before starting each opioid was significantly higher in the tapentadol group than those in the hydromorphone, oxycodone, and fentanyl groups and comparable to that in the methadone group. In patients without numbness, no significant differences were observed in decreases of the NRS scores on day seven among the five groups.

**Conclusions:**

Tapentadol and methadone may be more effective than hydromorphone, oxycodone, and fentanyl for cancer pain due to spinal metastasis with numbness.

## Background

Cancer-associated pain observed in many patients with advanced cancer has physical and psychological impacts, insulting the activities of daily living (ADL) and quality of life (QOL).^1^ The most common pain seen in patients with advanced cancer is cancer-associated bone pain, shown in 80% of patients with metastatic cancer.^2^ Bone metastasis pain is brief and excruciating that occurs after weight bearing or movement of involved portions of the skeleton, in addition to dull, constant, throbbing pain.^3,4^

Release of inflammatory substances such as endothelin, prostaglandins, and nerve growth factor from stromal/tumor cells induces sensitization and sensory nerves' excitation, causing nociceptive pain.^5,6^ Neuropathic pain can result from cancer-induced damage to the sensory nerves caused by infiltration and/or compression by the tumor cells and stretching or denervation as the bone expands and degrades.^7^ As bone metastases progress, nerves projecting to the bone marrow, cortical bone, and periosteal sensory nerves become involved in pain. Periosteum being a densely innervated tissue, activation of periosteal sensory nerves by mechanical stretching of the periosteum results in more intense pain. Thus, bone metastasis pain includes both inflammatory and neuropathic pain.^8^ Moreover, in the presynaptic neurons of the spinal dorsal horn, which bear approximately 75% of the spinal μ-opioid receptors, the expression of these receptors is reduced following nerve injury.^9^ Therefore, opioids are generally less effective in neuropathic pain than in inflammatory pain.^10,11^ A previous study reported that treating bone metastasis pain with increasing opioid doses did not provide adequate analgesia in some patients.^12^

Only half of the patients with bone metastasis pain experienced temporary pain relief from conventional medications, indicating the need for better analgesics.^13,14^

Unlike other opioids, tapentadol has μ-opioid receptor-stimulating and noradrenaline reuptake inhibitory effects, whereas methadone has μ-opioid receptor-stimulating and N-methyl-d-aspartate (NMDA) receptor antagonist effects. These multiaction opioids can effectively treat bone metastasis pain, including neuropathic pain.^15,16^ However, no previous study has been conducted to compare the efficacy of opioids in treating bone metastasis pain.

## Objective

This study aimed to gain insight into the optimal opioid selection for cancer pain due to spinal metastasis by comparing the efficacy and tolerability of tapentadol and methadone with other opioids for back pain caused by spinal metastasis, which are most frequent in bone metastases.^17^

## Design

The following data were collected from medical records: age, sex, body mass index, Eastern Cooperative Oncology Group performance status, Barthel Index, primary cancer site, pretreatment opioids and their morphine-equivalent daily dose (MEDD), the presence of pathological fracture at the spinal metastasis site, numbness, irradiation history for relieving spinal metastasis pain, concomitant medications, and laboratory values at the start of each opioid administration. The MEDD of pretreatment opioids was calculated using an equianalgesic dose table proposed by the National Comprehensive Cancer Network. The presence or absence of numbness was determined according to the medical records by physicians at the start of treatment with each opioid. Adverse events were evaluated according to the patients' complaints and medical records by physicians, nurses, and pharmacists.

Pain intensity, as reported by the patients, was based on the numerical rating scale (NRS) and obtained before and after each opioid administration (on days 0, 1, 2, 3, 5, 7, and 14). Bone metastasis pain is generally incidental and induced after movement.^3,4^ Therefore, this study investigated the worst pain in the past 24 hours included as an item in the Brief Pain Inventory Short Form (0 = no pain, 10 = pain as bad as the patient could imagine). This study also investigated usage count of opioid rescue doses before and after each opioid administration because the use of opioid rescue doses can affect changes in pain intensity.

The rate of improvement in the following movements within seven days after starting each opioid was investigated according to the patients' complaints and medical records by physicians, nurses, pharmacists, physiotherapists, and occupational therapists: walking without support, walking with support (using assistive devices; canes and walkers), stair climbing, standing up, maintaining steady upright standing, maintaining sitting without support, rising from a bed, repositioning without support, sleeping well at night, taking a bath without support, taking a bath with support, shopping in the hospital setting, and discharging from the hospital or going out for several hours. Walking with and without support was considered to improve when walking speed and distance increased after starting each opioid. Stair climbing, standing up, rising from a bed, and repositioning without support were considered to improve when the frequency and speed of each movement increased after starting each opioid. Maintaining steady upright standing and maintaining sitting without support were considered to improve when the time to hold each posture increased after starting each opioid.

Sleeping well at night was considered to improve when patients determined that their nighttime sleep improved after starting each opioid among patients who had nocturnal awakenings due to pain. Taking a bath without support, taking a bath with support, shopping in the hospital setting, and discharging from the hospital or going out for several hours were considered to improve when patients performed each event after starting each opioid among patients who wanted but were unable to perform each event due to pain.

## Settings/Subjects

This study enrolled Japanese inpatients who started a tapentadol extended-release tablet, methadone tablet, hydromorphone extended-release tablet, oxycodone extended-release tablet, or transdermal fentanyl patch for cancer pain due to spinal metastasis in Japan from January 1, 2013 to October 31, 2021. Spinal metastasis was determined by the physicians using magnetic resonance imaging. Since using other analgesics or bone-modifying agents (BMAs) may influence the efficacy of opioids for pain caused by spinal metastasis, 2, 3, 4, 4, and 8 patients, who started using other analgesics or BMAs within 7 days before initiating opioid therapy or within 14 days after initiating tapentadol, methadone, hydromorphone, oxycodone, and fentanyl, respectively, were excluded.

## Measurements

The primary endpoint was the difference in the NRS scores before and seven days after each opioid administration. The secondary endpoints were the differences in the NRS scores before and 3, 5, and 14 days after each opioid administration; the differences in the usage counts of opioid rescue doses before and 3, 5, 7, and 14 days after each opioid administration; the improvement rates for each movement within 7 days after starting each opioid; and the discontinuation rate of each opioid due to adverse events within 14 days after starting each opioid. Bone metastasis pain includes inflammatory and neuropathic pain.^8^ Therefore, this study compared the differences in the NRS scores, the differences in the usage counts of opioid rescue doses, the improvement rates for each movement, and the discontinuation rate among tapentadol, methadone, hydromorphone, oxycodone, and fentanyl groups after dividing patients according to the presence or absence of numbness, a typical symptom of neuropathic pain.^18^

## Statistics

The differences in the NRS scores and the usage counts of opioid rescue doses were analyzed using Dunnett's test. A chi-square for independence test was conducted to compare the improvement rates for each movement and the discontinuation rate of each opioid due to adverse events among the five groups.

The required sample size was determined to detect an effect size of 0.80 in the NRS scores on days 0 and 7 using a sample size calculator for a paired *t*-test referencing the sample size calculation in a previous study comparing the efficacy of opioids for cancer pain.^19^ Assuming a statistical probability at a 5% level with a power of 0.80, the minimum sample size was 15. All statistical analyses were performed using BellCurve for Excel (Social Survey Research Information Co., Ltd., Tokyo, Japan); *p*-values of <0.05 were considered statistically significant.

## Ethics Approval

This study was conducted in accordance with the ethical principles for medical research outlined in the Declaration of Helsinki (1964) and approved by the Ethics Review Board of the Osaka University Graduate School of Pharmaceutical Sciences (IRB Approval Code No. 2021-12). Written consent for publication of this study was obtained from all patients.

## Results

The data of 52, 53, 60, 51, and 55 patients were included in the tapentadol, methadone, hydromorphone, oxycodone, and fentanyl groups, respectively. Table 1 lists the baseline characteristics of the patients. The MEDD of pretreatment opioids was significantly higher in the methadone group than in the tapentadol, hydromorphone, oxycodone, and fentanyl groups (*p* <0.001). No significant differences were observed for the other baseline characteristics among the five groups. Supplementary Table S1 shows the dose of each opioid and its morphine-equivalent daily dose.

**Table 1.** Baseline Patient Characteristics

	Tapentadol (***n*** = 52)	Methadone (***n*** = 53)	Hydromorphone (***n*** = 60)	Oxycodone (***n*** = 51)	Fentanyl (***n*** = 55)	***p***
Age (years), mean ± SD (range)	72.8 ± 10.6 (47–91)	70.5 ± 10.4 (49–87)	74.1 ± 11.7 (43–94)	70.1 ± 12.1 (43–91)	74.9 ± 11.9 (47–93)	0.07^a^
Sex, male, *n* (%)	20 (38.5)	20 (37.7)	22 (36.7)	22 (43.2)	25 (45.5)	0.86^b^
BMI (kg/m^2^), mean ± SD (range)	20.1 ± 3.9 (12.3–31.7)	19.0 ± 3.0 (15.5–29.2)	19.2 ± 3.5 (13.1–29.7)	19.5 ± 3.2 (12.4–27.2)	18.8 ± 3.5 (12.3–25.9)	0.49^a^
ECOG PS, *n* (%)						
4	11 (21.2)	11 (20.8)	11 (18.3)	6 (11.8)	16 (29.1)	0.31^b^
3	15 (28.8)	19 (35.8)	17 (28.3)	17 (33.3)	22 (40.0)	
2	11 (21.2)	13 (24.5)	11 (18.3)	9 (17.6)	10 (18.2)	
≤1	15 (28.8)	10 (18.9)	21 (35.0)	19 (37.3)	7 (12.7)	
BI, *n* (%)						
0–25	8 (15.4)	8 (15.1)	10 (16.7)	6 (11.8)	9 (16.4)	0.98^b^
26–50	11 (21.2)	10 (18.9)	12 (20.0)	9 (17.6)	6 (10.9)	
51–75	17 (32.7)	16 (30.2)	18 (30.0)	17 (33.3)	22 (40.0)	
76–100	16 (30.8)	19 (35.8)	20 (33.3)	19 (37.3)	18 (32.7)	
Primary cancer site, *n* (%)						
Lung	16 (30.8)	18 (34.0)	15 (25.0)	14 (27.5)	16 (29.1)	N.A.
Blood	9 (17.3)	6 (11.3)	8 (13.3)	8 (15.7)	6 (10.9)	
Breast	7 (13.5)	5 (9.4)	6 (10.0)	7 (13.7)	8 (14.5)	
Prostate	6 (11.5)	3 (5.7)	5 (8.3)	4 (7.8)	4 (7.3)	
Colon	4 (7.7)	5 (9.4)	4 (6.7)	5 (9.8)	4 (7.3)	
Pancreas	4 (7.7)	2 (3.8)	8 (13.3)	3 (5.9)	4 (7.3)	
Others	6 (11.5)	14 (26.4)	14 (23.3)	10 (19.6)	13 (23.6)	
Pretreatment opioids, *n* (%)						
Oxycodone	14 (26.9)	16 (30.2)	20 (33.3)	—	25 (45.5)	N.A.
Fentanyl	12 (23.1)	14 (26.4)	12 (20.0)	14 (27.5)	—	
Hydromorphone	10 (19.2)	11 (20.8)	—	8 (15.7)	6 (10.9)	
Morphine	3 (5.8)	6 (11.3)	10 (16.7)	9 (17.6)	6 (10.9)	
Tapentadol	—	5 (9.4)	6 (10.0)	3 (5.9)	4 (7.3)	
Tramadol	9 (17.3)	1 (1.9)	7 (11.7)	9 (17.6)	8 (14.5)	
Naive	4 (7.7)	0 (0.0)	5 (8.3)	8 (15.7)	6 (10.9)	
MEDD of pretreatment opioids (mg/day), mean ± SD (range)	33.7 ± 61.3 (0–390)	95.5 ± 69.1 (7.5–320)	22.7 ± 58.1 (0–390)	32.7 ± 57.6 (0–360)	28.6 ± 33.7 (0–380)	<0.001^a^
With pathologic fracture, *n* (%)	22 (42.3)	20 (37.7)	18 (30.0)	21 (41.2)	19 (34.5)	0.66^b^
With numbness, *n* (%)	36 (69.2)	37 (69.8)	40 (66.7)	35 (68.6)	33 (60.0)	0.81^b^
With a history of irradiation, *n* (%)	8 (15.4)	13 (24.5)	14 (23.3)	10 (19.6)	13 (23.6)	0.77^b^
Concomitant medications, *n* (%) (including duplicate answers)						
Acetaminophen	10 (19.2)	12 (22.6)	13 (21.7)	13 (25.5)	12 (21.8)	0.96^b^
NSAIDs						
Celecoxib	12 (23.1)	8 (15.1)	8 (13.3)	9 (17.6)	13 (23.6)	0.53^b^
Loxoprofen	5 (9.6)	10 (18.9)	8 (13.3)	7 (13.7)	7 (12.7)	0.74^b^
Naproxen	4 (7.7)	3 (5.7)	4 (6.7)	3 (5.9)	2 (3.6)	0.93^b^
Others	2 (3.8)	3 (5.7)	1 (1.7)	1 (2.0)	5 (9.1)	0.30^b^
None	30 (57.7)	31 (58.5)	39 (65.0)	34 (66.7)	29 (52.7)	0.57^b^
Adjuvant analgesics						
Corticosteroids	14 (26.9)	21 (39.6)	21 (35.0)	15 (29.4)	21 (38.2)	0.58^b^
Gabapentinoids	8 (15.4)	8 (15.1)	9 (15.0)	12 (23.5)	10 (18.2)	0.75^b^
SNRI	5 (9.6)	4 (7.5)	1 (1.7)	1 (2.0)	6 (10.9)	0.14^b^
Others	2 (3.8)	4 (7.5)	1 (1.7)	2 (3.9)	0 (0.0)	0.24^b^
None	25 (48.1)	21 (39.6)	32 (53.5)	27 (52.9)	26 (47.3)	0.61^b^
BMAs						
Zoledronic acid	4 (7.7)	5 (9.4)	5 (8.3)	6 (11.8)	4 (7.3)	0.93^b^
Denosumab	2 (3.8)	1 (1.9)	1 (1.7)	0 (0.0)	2 (3.6)	0.66^b^
None	46 (88.5)	47 (88.7)	54 (90.0)	45 (88.2)	49 (89.1)	0.99^b^
Laboratory values at the start of each opioid administration, median (IQR)						
AST (U/L)	25.5 (8.0–160.0)	22.0 (10.0–125.0)	21.0 (7.0–175.0)	31.0 (10.0–150.0)	25.0 (9.0–163.0)	0.13^c^
ALT (U/L)	14.0 (5.0–63.0)	15.0 (6.0–65.0)	15.0 (5.0–49.0)	16.0 (5.0–98.0)	15.5 (5.0–97.0)	0.73^c^
γ-GTP (U/L)	39.0 (10.0–934.0)	51.0 (7.0–768.0)	49.5 (7.0–1041.0)	43.5 (11.0–931.0)	62.0 (12.0–1144.0)	0.67^c^
Scr (mg/dL)	0.67 (0.30–1.43)	0.58 (0.20–2.12)	0.80 (0.39–2.08)	0.69 (0.38–1.75)	0.67 (0.18–2.26)	0.23^c^
eGFR (mL/min)	71.8 (35.9–169.4)	78.0 (21.5–138.8)	64.1 (19.4–133.1)	70.4 (30.0–148.1)	70.5 (16.5–239.7)	0.55^c^
BUN (mg/dL)	16.2 (5.4–50.6)	14.4 (5.4–47.9)	17.3 (5.3–65.9)	14.5 (5.9–51.6)	17.7 (4.8–60.6)	0.32^c^

^a^ Single-factor ANOVA.

^b^ Chi-square for independence test.

^c^ Kruskal–Wallis test.

ALT, alanine transaminase; AST, aspartate transaminase; BI, Barthel Index; BMAs, bone-modifying agents; BMI, body mass index; BUN, blood urea nitrogen; ECOG PS, Eastern Cooperative Oncology Group Performance Status; eGFR, estimated glomerular filtration rate; γ-GTP, γ-glutamyl transpeptidase; IQR, interquartile range; MEDD, morphine-equivalent daily dose; N.A., not available; NSAIDs, nonsteroidal anti-inflammatory drugs; Scr, serum creatinine; SD, standard deviation; SNRI, serotonin–norepinephrine reuptake inhibitor.

**FIG. 1.**
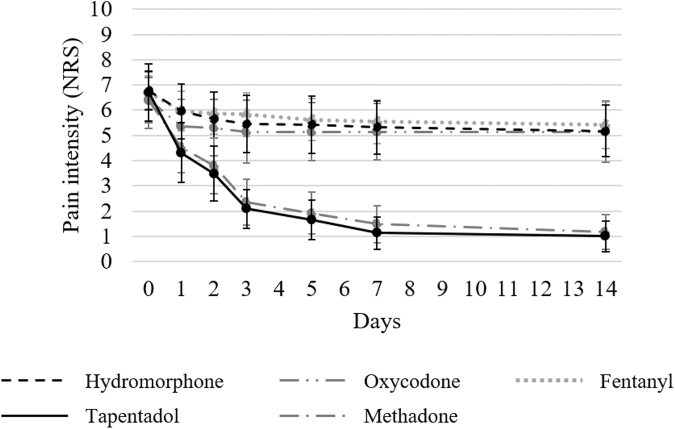
Change in the mean NRS scores on pain in patients with numbness. The NRS scores were investigated at baseline and after administering each opioid (on days 0, 1, 2, 3, 5, 7, and 14) in patients with numbness. NRS, numerical rating scale.

**FIG. 2.**
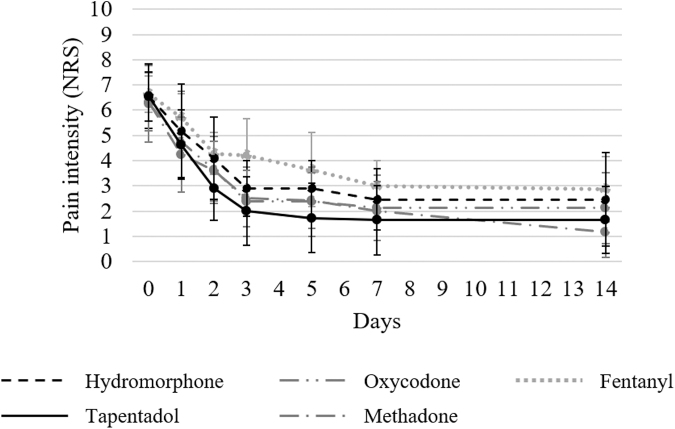
Change in the mean NRS scores on pain in patients without numbness. The NRS scores were investigated at baseline and after administering each opioid (on days 0, 1, 2, 3, 5, 7, and 14) in patients without numbness.

The changes in the worst pain scores (NRS) in the past 24 hours before and after each opioid administration in patients with and without numbness are shown in Figures 1 and 2, respectively. No significant differences were observed in the NRS scores before each opioid administration among the five groups in both patients with numbness (*p* = 0.96) and those without numbness (*p* = 0.99). In all groups, the NRS scores decreased until day 14. Table 2 presents the decrease in the NRS scores on days 1, 2, 3, 5, 7, and 14 compared with before starting each opioid. The tapentadol group significantly outperformed hydromorphone, oxycodone, and fentanyl groups in decreasing NRS scores in patients with numbness compared with before starting each opioid on all evaluation days. In patients without numbness, no significant differences were observed in decreases of the NRS scores between the tapentadol and hydromorphone, oxycodone, and fentanyl groups on all evaluation days. However, in patients with and without numbness, comparisons of decreases in the NRS scores between the tapentadol and methadone groups revealed no significant differences on all evaluation days.

**Table 2.** Decreases of the Numerical Rating Scale Scores Compared to Before Starting Each Opioid

	Day 1	Day 2	Day 3	Day 5	Day 7	Day 14
Patients with numbness						
Tapentadol (*n* = 36), mean ± SD	2.39 ± 2.19	3.22 ± 2.35	4.61 ± 2.37	5.04 ± 2.50	5.57 ± 2.87	5.70 ± 2.98
Methadone (*n* = 37), mean ± SD	1.87 ± 1.66	2.61 ± 1.37	4.04 ± 2.29	4.48 ± 2.56	4.91 ± 2.66	5.22 ± 2.70
Hydromorphone (*n* = 40), mean ± SD	0.82 ± 1.40	1.14 ± 1.39	1.32 ± 1.73	1.36 ± 1.73	1.45 ± 1.57	1.59 ± 1.50
Oxycodone (*n* = 35), mean ± SD	1.05 ± 1.64	1.10 ± 1.86	1.25 ± 1.80	1.25 ± 1.77	1.25 ± 1.62	1.25 ± 1.71
Fentanyl (*n* = 33), mean ± SD	0.75 ± 1.26	0.83 ± 1.20	0.88 ± 1.45	1.08 ± 1.67	1.17 ± 1.61	1.29 ± 1.85
*p* (tapentadol vs. methadone)	0.67	0.55	0.73	0.77	0.70	0.88
*p* (tapentadol vs. hydromorphone)	<0.001	<0.001	<0.001	<0.001	<0.001	<0.001
*p* (tapentadol vs. oxycodone)	<0.001	<0.001	<0.001	<0.001	<0.001	<0.001
*p* (tapentadol vs. fentanyl)	<0.001	<0.001	<0.001	<0.001	<0.001	<0.001
*p* (methadone vs. hydromorphone)	0.22	0.033	<0.001	<0.001	<0.001	<0.001
*p* (methadone vs. oxycodone)	0.49	0.033	<0.001	<0.001	<0.001	<0.001
*p* (methadone vs. fentanyl)	0.15	0.004	<0.001	<0.001	<0.001	<0.001
Patients without numbness						
Tapentadol (*n* = 16), mean ± SD	1.91 ± 2.21	3.64 ± 2.16	4.55 ± 2.88	4.82 ± 2.64	4.91 ± 2.59	4.91 ± 2.51
Methadone (*n* = 16), mean ± SD	2.08 ± 2.57	2.75 ± 2.42	3.83 ± 2.55	3.92 ± 2.47	4.33 ± 2.67	5.17 ± 3.35
Hydromorphone (*n* = 20), mean ± SD	1.36 ± 1.96	2.45 ± 2.16	3.64 ± 1.69	3.64 ± 1.69	4.09 ± 2.30	4.09 ± 2.70
Oxycodone (*n* = 16), mean ± SD	1.50 ± 2.14	2.63 ± 2.50	3.88 ± 3.60	3.88 ± 3.60	4.13 ± 3.48	4.13 ± 3.44
Fentanyl (*n* = 22), mean ± SD	0.93 ± 1.44	2.36 ± 1.34	2.43 ± 2.24	3.00 ± 2.08	3.64 ± 1.50	3.79 ± 2.26
*p* (tapentadol vs. methadone)	0.99	0.70	0.91	0.80	0.95	0.99
*p* (tapentadol vs. hydromorphone)	0.93	0.49	0.82	0.63	0.85	0.90
*p* (tapentadol vs. oxycodone)	0.98	0.69	0.95	0.83	0.90	0.94
*p* (tapentadol vs. fentanyl)	0.59	0.37	0.14	0.22	0.52	0.72
*p* (methadone vs. hydromorphone)	0.92	0.99	0.99	0.99	0.99	0.89
*p* (methadone vs. oxycodone)	0.97	0.99	0.99	0.99	0.99	0.93
*p* (methadone vs. fentanyl)	0.62	0.99	0.64	0.88	0.95	0.73

Dunnett's test.

**FIG. 3.**
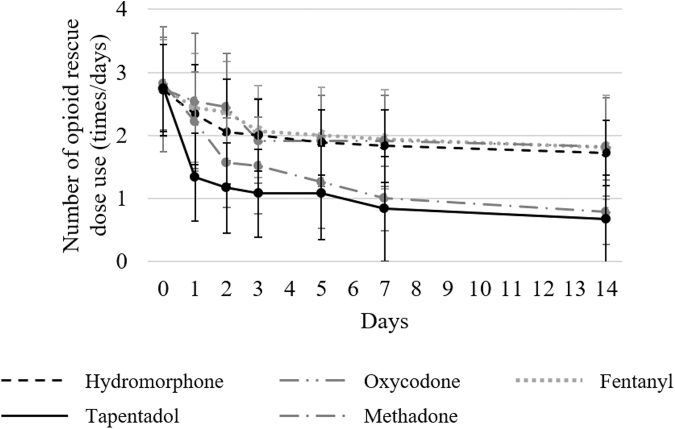
Change in the mean number of opioid rescue doses use in patients with numbness. The usage counts of rescue doses were investigated before and after administering each opioid (on days 0, 1, 2, 3, 5, 7, and 14) in patients with numbness. When multiple types of rescue doses were used, the total usage counts were determined.

**FIG. 4.**
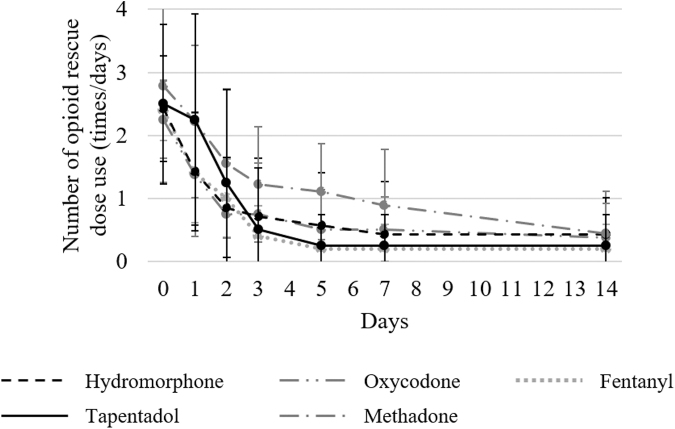
Change in the mean number of opioid rescue doses use in patients without numbness. The usage counts of rescue doses were investigated before and after administering each opioid (on days 0, 1, 2, 3, 5, 7, and 14) in patients without numbness. When multiple types of rescue doses were used, the total usage counts were determined.

The changes in the usage counts of opioid rescue doses before and after each opioid administration in patients with and without numbness are displayed in Figures 3 and 4, respectively. No significant differences in the usage counts of opioid rescue doses before each opioid administration were observed among the five groups in both patients with numbness (*p* = 0.99) and those without numbness (*p* = 0.97). In all groups, the usage counts of opioid rescue doses decreased until day 14. Table 3 shows the decrease in the usage counts of opioid rescue doses compared with before starting each opioid and Supplementary Table S2 shows the morphine-equivalent daily dose of opioid rescue doses. In patients with numbness, a decrease in the usage counts of opioid rescue doses compared with before starting each opioid was significantly greater in the tapentadol group than those in the other groups on day one. On day two, a decrease in the usage counts of opioid rescue doses compared with before starting each opioid was significantly higher in the tapentadol group than those in the hydromorphone, oxycodone, and fentanyl groups as on day one.

**Table 3.** Decreases of the Usage Counts of Opioid Rescue Doses Compared With Before Starting Each Opioid

	Day 1	Day 2	Day 3	Day 5	Day 7	Day 14
Patients with numbness						
Tapentadol (*n* = 36), mean ± SD	1.42 ± 1.24	1.58 ± 1.24	1.67 ± 0.89	1.67 ± 1.30	1.92 ± 1.16	2.08 ± 1.00
Methadone (*n* = 37), mean ± SD	0.61 ± 1.12	1.26 ± 1.42	1.30 ± 1.46	1.57 ± 1.73	1.83 ± 1.59	2.04 ± 1.52
Hydromorphone (*n* = 40), mean ± SD	0.39 ± 0.70	0.67 ± 0.77	0.72 ± 0.96	0.83 ± 0.99	0.89 ± 1.32	1.00 ± 1.24
Oxycodone (*n* = 35), mean ± SD	0.18 ± 0.40	0.27 ± 0.47	0.82 ± 0.98	0.82 ± 0.98	0.82 ± 1.25	0.91 ± 1.22
Fentanyl (*n* = 33), mean ± SD	0.31 ± 0.70	0.38 ± 0.62	0.69 ± 1.25	0.75 ± 1.24	0.81 ± 1.33	0.94 ± 1.39
*p* (tapentadol vs. methadone)	0.047	0.77	0.78	0.99	0.99	0.99
*p* (tapentadol vs. hydromorphone)	0.011	0.047	0.10	0.26	0.14	0.10
*p* (tapentadol vs. oxycodone)	0.006	0.011	0.24	0.34	0.17	0.11
*p* (tapentadol vs. fentanyl)	0.007	0.010	0.10	0.21	0.12	0.08
*p* (methadone vs. hydromorphone)	0.94	0.36	0.52	0.41	0.20	0.10
*p* (methadone vs. oxycodone)	0.70	0.77	0.79	0.55	0.28	0.15
*p* (methadone vs. fentanyl)	0.85	0.72	0.50	0.34	0.17	0.09
Patients without numbness						
Tapentadol (*n* = 16), mean ± SD	0.25 ± 0.50	1.25 ± 1.89	2.00 ± 1.83	2.25 ± 1.50	2.25 ± 1.50	2.25 ± 1.50
Methadone (*n* = 16), mean ± SD	0.56 ± 0.73	1.22 ± 1.48	1.56 ± 1.94	1.67 ± 1.50	1.89 ± 1.69	2.33 ± 2.29
Hydromorphone (*n* = 20), mean ± SD	1.00 ± 1.53	1.57 ± 1.40	1.71 ± 1.60	1.86 ± 1.46	2.00 ± 1.41	2.00 ± 1.41
Oxycodone (*n* = 16), mean ± SD	0.88 ± 1.13	1.50 ± 0.93	1.50 ± 0.93	1.75 ± 0.89	1.75 ± 1.04	1.88 ± 0.64
Fentanyl (*n* = 22), mean ± SD	1.00 ± 0.71	1.40 ± 0.89	2.00 ± 0.71	2.20 ± 0.84	2.20 ± 0.84	2.20 ± 0.45
*p* (tapentadol vs. methadone)	0.95	0.99	0.95	0.83	0.97	0.99
*p* (tapentadol vs. hydromorphone)	0.55	0.98	0.99	0.95	0.99	0.99
*p* (tapentadol vs. oxycodone)	0.67	0.99	0.94	0.89	0.91	0.98
*p* (tapentadol vs. fentanyl)	0.61	0.99	0.99	0.99	0.99	0.99
*p* (methadone vs. hydromorphone)	0.91	0.98	0.99	0.99	0.99	0.99
*p* (methadone vs. oxycodone)	0.97	0.99	0.99	0.99	0.99	0.97
*p* (methadone vs. fentanyl)	0.94	0.99	0.98	0.94	0.99	0.99

Dunnett's test.

After day three, no significant differences in decreases in the usage counts of opioid rescue doses were observed among the five groups of patients with numbness. However, in patients without numbness, no significant differences in decreases in the usage counts of opioid rescue doses were observed among the five groups on all evaluation days. In patients with and without numbness, no significant differences in the MEDD of opioid rescue doses were observed among all groups at baseline and after administering each opioid.

Table 4 displays the improvement rate for each movement within seven days after starting each opioid in patients with and without numbness. In patients with numbness, the improvement rates for all movements within seven days after starting each opioid were higher in the tapentadol and methadone groups than those in the hydromorphone, oxycodone, and fentanyl groups. Meanwhile, in patients without numbness, no significant differences were observed in improvement rates for any movements among the five groups.

**Table 4.** Improvement Rates for Each Movement Within Seven Days After Starting Each Opioid

	Tapentadol,***n*** (%)	Methadone,***n*** (%)	Hydromorphone, ***n*** (%)	Oxycodone,***n*** (%)	Fentanyl,***n*** (%)	***p***
Patients with numbness						
Walking without support	20/22 (90.9)	17/19 (89.5)	7/25 (28.0)	5/22 (22.7)	4/15 (26.7)	<0.001
Walking with support	15/18 (83.3)	11/14 (78.6)	5/25 (20.0)	3/18 (16.7)	3/20 (15.0)	<0.001
Stair climbing	17/21 (81.0)	14/20 (70.0)	5/20 (25.0)	3/21 (14.3)	4/17 (23.5)	<0.001
Standing up	27/32 (84.4)	17/22 (77.3)	7/28 (25.0)	4/22 (18.2)	7/28 (25.0)	<0.001
Maintaining steady upright standing	24/27 (88.9)	13/17 (76.5)	6/27 (22.2)	4/22 (18.2)	7/27 (25.9)	<0.001
Maintaining sitting without support	21/24 (87.5)	17/22 (77.3)	9/22 (40.9)	7/20 (35.0)	7/18 (38.9)	<0.001
Rising from a bed	24/29 (82.8)	19/26 (73.1)	9/29 (31.0)	7/23 (30.4)	7/22 (31.8)	<0.001
Repositioning without support	21/22 (95.5)	17/19 (89.5)	4/22 (18.2)	4/22 (18.2)	4/21 (19.0)	<0.001
Sleeping well at night	30/31 (96.8)	32/34 (94.1)	11/28 (39.3)	11/32 (34.4)	8/26 (30.8)	<0.001
Taking a bath without support	22/24 (91.7)	19/20 (95.0)	14/26 (53.8)	13/25 (52.0)	12/23 (52.2)	<0.001
Taking a bath with support	11/13 (84.6)	14/17 (82.4)	7/18 (38.9)	5/15 (33.3)	8/21 (38.1)	0.002
Shopping in the hospital setting	27/32 (84.4)	25/32 (78.1)	12/35 (34.3)	8/33 (24.2)	11/32 (34.4)	<0.001
Discharging from the hospital or going out for several hours	22/27 (81.5)	19/27 (70.4)	13/33 (39.4)	9/30 (30.0)	12/32 (37.5)	<0.001
Patients without numbness						
Walking without support	8/9 (88.9)	8/9 (88.9)	9/13 (69.2)	7/13 (53.8)	9/14 (64.3)	0.29
Walking with support	10/10 (100.0)	8/9 (88.9)	7/12 (58.3)	6/11 (54.5)	7/11 (63.6)	0.08
Stair climbing	7/8 (87.5)	7/9 (77.8)	5/12 (41.7)	4/10 (40.0)	7/14 (50.0)	0.13
Standing up	11/12 (91.7)	12/13 (92.3)	11/15 (73.3)	9/14 (64.3)	14/19 (73.7)	0.31
Maintaining steady upright standing	11/11 (100.0)	12/13 (92.3)	11/16 (68.8)	10/15 (66.7)	12/17 (70.6)	0.13
Maintaining sitting without support	12/12 (100.0)	8/9 (88.9)	11/14 (78.6)	8/11 (72.7)	13/17 (76.5)	0.39
Rising from a bed	13/14 (92.9)	14/16 (87.5)	11/16 (68.8)	8/12 (66.7)	8/13 (61.5)	0.21
Repositioning without support	11/11 (100.0)	10/10 (100.0)	11/15 (73.3)	8/12 (66.7)	11/15 (73.3)	0.10
Sleeping well at night	13/13 (100.0)	14/14 (100.0)	15/18 (83.3)	12/15 (80.0)	14/17 (82.4)	0.23
Taking a bath without support	11/11 (100.0)	9/10 (90.0)	8/11 (72.7)	8/13 (61.5)	10/15 (66.7)	0.14
Taking a bath with support	8/8 (100.0)	9/9 (100.0)	8/12 (66.7)	6/9 (66.7)	9/13 (69.2)	0.13
Shopping in the hospital setting	14/14 (100.0)	15/16 (93.8)	11/15 (73.3)	10/14 (71.4)	15/20 (75.0)	0.13
Discharging from the hospital or going out for several hours	9/10 (90.0)	8/10 (80.0)	7/11 (63.6)	6/10 (60.0)	8/14 (57.1)	0.40

Chi-square for independence test.

No patient discontinued tapentadol due to adverse events within 14 days after starting tapentadol. One patient (1.9%) discontinued methadone due to nausea. Five patients (8.1%) and two patients (3.2%) discontinued hydromorphone due to nausea and daytime sleepiness, respectively. Three patients (5.9%), three patients (5.9%), and one patient (2.0%) discontinued oxycodone due to constipation, nausea, and delirium, respectively. Two patients (3.6%), two patients (3.6%), and one patient (1.8%) discontinued fentanyl due to daytime sleepiness, pruritus, and nausea, respectively.

## Discussion

To our knowledge, this is the first study to compare the effects of opioids on cancer pain due to spinal metastasis. In patients with numbness, both a decrease of the NRS scores on day seven compared with baseline and the improvement rate for all movements within seven days after starting the administration were higher in the tapentadol group than those in the hydromorphone, oxycodone, and fentanyl groups and comparable to those in the methadone group. These results suggest that tapentadol and methadone may be more effective than hydromorphone, oxycodone, and fentanyl for cancer pain due to spinal metastasis with numbness.

Recently, it has become clear that the noradrenaline pathway is important in preventing the progression of neuropathic pain. This endogenous descending pain modulating system plays a key role in shaping the spatial and temporal expressions of the neuropathic pain phenotype following nerve injury.^20^ Namely, the baseline pain sensitivity is only a little influenced by the noradrenergic system, whereas under neuropathic pain, the central and peripheral noradrenergic systems are subject to plastic changes that mitigate against antinociceptive efficacy.^21^ Tapentadol with noradrenaline reuptake inhibition increases noradrenaline in the spinal cord and activates descending inhibitory pain pathways.^22^ Interestingly, experimental and clinical evidence indicates that the noradrenaline reuptake inhibition component of tapentadol may become predominant compared with μ-opioid receptor-stimulating component in neuropathic conditions.^9,22^ In this study, among patients with numbness, a decrease of the NRS scores before and after starting the administration was significantly greater in patients administered tapentadol than hydromorphone, oxycodone, and fentanyl groups, which have only a μ-opioid receptor-stimulating effect and made the NRS scores little reduced. Thus, tapentadol may be suitable for spinal metastasis pain, especially neuropathic pain.

Moreover, methadone with NMDA receptor antagonism also showed a significantly higher analgesic effect for cancer pain due to spinal metastasis with numbness in this study than hydromorphone, oxycodone, and fentanyl. Glutamate released from cancer cells that give rise to bone cancer excites and induces skeletal and cutaneous hyperalgesia and mechanical sensitization by potentially activating peripherally expressed NMDA receptors.^23^ NMDA receptor antagonists have been reported effective for such neuropathic pain,^24^ and the results of this study were consistent with these previous studies. In the methadone group of this study, the MEDD of pretreatment opioids was significantly higher than in the other groups. Therefore, some of the NRS reduction in the methadone group might be due to its effect on opioid-induced hyperalgesia through NMDA antagonism.

In patients with numbness, the improvement rates for each movement within seven days after starting the administration were larger in the tapentadol and methadone groups than those in the hydromorphone, oxycodone, and fentanyl groups in this study. Bone metastasis pain is commonly induced after movement, and a previous study suggested that skeletal-related events such as bone metastasis exerted a serious impact on QOL and ADL in the patients.^25^ Additionally, being bedridden for a long time due to pain increases the risk of deep venous thrombosis, bedsores, and delirium due to hypercalcemia.^26^ Therefore, this study clarified the efficacy of tapentadol and methadone for spinal metastasis pain could be clinically significant for pain management and in preventing complications associated with these prolonged rests. The ADL level reportedly affects the availability of discharge in patients with spinal bone metastases.^27^

In this study, the improvement rates for discharge from the hospital or going out for several hours in the tapentadol and methadone groups, which had higher improvement rates for all movements, were also significantly higher than those in the hydromorphone, oxycodone, and fentanyl groups. This study is also significant for discharge support because discharge from the hospital and going out for several hours are important options for cancer patients and can be goals for patients and their families, especially at the end of life.

In a previous study of patients with bone lesions related to multiple myeloma, >70% of patients were reported to have neuropathic pain components.^15^ Approximately 70% of patients in this study also had numbness, a typical neuropathic pain symptom. Both decreases of the NRS scores and improvement rates for all movements were higher in the tapentadol and methadone group than those in the hydromorphone, oxycodone, and fentanyl groups in patients with numbness, although no statistically significant differences were observed in decreases of the NRS scores and improvement rates for any movements among the five groups in patients without numbness. Therefore, numbness can be a prime indicator in selecting the optimal therapeutic medication for managing spinal metastasis pain.

In patients without numbness, the use of opioid rescue doses decreased to <1 time/day within 14 days in all groups. In patients with numbness, the use of opioid rescue doses decreased in all groups, and those in the tapentadol and methadone group tended to be larger than those in the other three groups, like decreases in the NRS scores. This result suggested that the differences in decreases of the NRS scores among the five groups could be due to the analgesic effects of the opioids rather than the analgesic effects of the opioid rescue doses. Moreover, tapentadol was shown to have fewer rescue opioid doses in the first one to two days after initiation. This could be related to the shorter half-life of tapentadol; therefore, it might reach a steady state earlier than other opioids.

Regarding tolerability, no patient discontinued tapentadol due to adverse events; among the five groups, the discontinuation rate due to adverse events was the lowest in the tapentadol group. Gastrointestinal adverse events are well tolerated because tapentadol has a low affinity for the μ-opioid receptor.^28^ Tapentadol was associated with significantly lower incidences of constipation and vomiting and significant improvements in QOL measures compared with oxycodone/naloxone.^29^ Therefore, tapentadol is a viable treatment option even in patients at high risk of adverse events, such as the elderly.

This study had several limitations. First, this was a retrospective study. Although no significant differences were observed in the baseline patient characteristics other than MEDD of pretreatment opioids (Table 1), confounding for patients' backgrounds among the five groups may not be eliminated. Second, this study could not evaluate the types of pain with pain assessment scales specific to neuropathic pain. Therefore, validating our findings using assessment scales such as the painDETECT questionnaire is desirable.

## Conclusions

Tapentadol and methadone may be more effective than hydromorphone, oxycodone, and fentanyl for cancer pain due to spinal metastasis with numbness. As spinal metastasis pain significantly affects the ADL of patients, this study clarifying the optimal opioids for cancer pain due to spinal metastasis for the first time is clinically meaningful in pain and ADL improvement.

## Supplementary Material

Supplemental data

Supplemental data

## Abbreviations Used

ADL: activities of daily living
ALT: alanine transaminase
ANOVA: analysis of variance
AST: aspartate transaminase
BI: Barthel Index
BMAs: bone-modifying agents
BMI: body mass index
BUN: blood urea nitrogen
ECOG PS: Eastern Cooperative Oncology Group Performance Status
eGFR: estimated glomerular filtration rate
γ-GTP: γ-glutamyl transpeptidase
IQR: interquartile range
MEDD: morphine-equivalent daily dose
N.A.: not available
NMDA: N-methyl-d-aspartate
NRS: numerical rating scale
NSAIDs: nonsteroidal anti-inflammatory drugs
QOL: quality of life
Scr: serum creatinine
SD: standard deviation
SNRI: serotonin–norepinephrine reuptake inhibitor
