# Supplementary material for: Differences in the Analgesic Effect of Opioids on Pain in Cancer Patients With Spinal Metastases
Source: Palliat Med Rep. 2023 Aug 9;4(1):220–30. doi: 10.1089/pmr.2023.0018 (PMC10457616; doi:10.1089/pmr.2023.0018)
Supplement: Supplemental data [file Supp_TableS1.docx]

**Supplemental Table 1.** Doses of opioids and their morphine-equivalent daily doses

|  | Day 7 | | Day 14 | |
| --- | --- | --- | --- | --- |
|  | Dose of opioids | MEDD of opioids | Dose of opioids | MEDD of opioids |
| Patients with numbness |  |  |  |  |
| Tapentadol (n = 36), mean ± SD | 95.8 ± 66.9 | 28.8 ± 20.1 | 108.3 ± 68.1 | 32.5 ± 20.4 |
| Methadone (n = 37), mean ± SD | 19.3 ± 10.5 | – | 22.1 ± 15.3 | – |
| Hydromorphone (n = 40), mean ± SD | 6.00 ± 6.60 | 30.0 ± 33.0 | 8.00 ± 8.26 | 40.0 ± 41.3 |
| Oxycodone (n = 35), mean ± SD | 21.2 ± 22.1 | 31.8 ± 33.2 | 24.9 ± 25.0 | 37.3 ± 37.5 |
| Fentanyl (n = 33), mean ± SD | 1.10 ± 0.65 | 33.1 ± 19.5 | 1.45 ± 0.93 | 43.6 ± 27.9 |
| *p-value* (tapentadol vs. hydromorphone) | – | 0.99 | – | 0.64 |
| *p-value* (tapentadol vs. oxycodone) | – | 0.94 | – | 0.88 |
| *p-value* (tapentadol vs. fentanyl) | – | 0.85 | – | 0.37 |
| Patients without numbness |  |  |  |  |
| Tapentadol (n = 36), mean ± SD | 82.8 ± 52.2 | 24.8 ± 15.7 | 95.3 ± 57.9 | 28.6 ± 17.4 |
| Methadone (n = 37), mean ± SD | 16.4 ± 7.6 | – | 18.1 ± 10.2 | – |
| Hydromorphone (n = 40), mean ± SD | 4.48 ± 3.80 | 22.4 ± 19.0 | 4.80 ± 3.96 | 24.0 ± 19.8 |
| Oxycodone (n = 35), mean ± SD | 15.7 ± 15.1 | 23.6 ± 22.7 | 17.3 ± 16.5 | 26.0 ± 24.8 |
| Fentanyl (n = 33), mean ± SD | 0.80 ± 0.37 | 24.0 ± 11.1 | 0.94 ± 0.41 | 28.1 ± 12.4 |
| *p-value* (tapentadol vs. hydromorphone) | – | 0.95 | – | 0.80 |
| *p-value* (tapentadol vs. oxycodone) | – | 0.99 | – | 0.96 |
| *p-value* (tapentadol vs. fentanyl) | – | 0.99 | – | 0.99 |
| Dunnett's test.  Abbreviations: MEDD, morphine-equivalent daily dose; SD, standard deviation; vs., versus. | | | | |
