# Supplementary material for: Differences in the Analgesic Effect of Opioids on Pain in Cancer Patients With Spinal Metastases
Source: Palliat Med Rep. 2023 Aug 9;4(1):220–30. doi: 10.1089/pmr.2023.0018 (PMC10457616; doi:10.1089/pmr.2023.0018)
Supplement: Supplemental data [file Supp_TableS2.docx]

.

**Supplemental Table 2.** The morphine-equivalent daily dose of opioid rescue doses

|  | Day 0 | Day 3 | Day 5 | Day 7 | Day 14 |
| --- | --- | --- | --- | --- | --- |
| Patients with numbness |  |  |  |  |  |
| Tapentadol (n = 36), mean ± SD | 21.1 ± 12.3 | 9.2 ± 10.9 | 7.9 ± 8.4 | 7.1 ± 11.3 | 4.8 ± 9.2 |
| Methadone (n = 37), mean ± SD | 30.5 ± 39.0 | 21.2 ± 36.7 | 19.8 ± 39.3 | 12.5 ± 26.2 | 10.0 ± 25.8 |
| Hydromorphone (n = 40), mean ± SD | 21.0 ± 26.5 | 16.0 ± 17.9 | 15.9 ± 18.1 | 15.6 ± 12.9 | 14.7 ± 15.3 |
| Oxycodone (n = 35), mean ± SD | 22.2 ± 16.9 | 16.5 ± 13.7 | 16.5 ± 15.3 | 16.0 ± 18.8 | 15.4 ± 17.8 |
| Fentanyl (n = 33), mean ± SD | 22.0 ± 17.3 | 16.9 ± 14.8 | 16.0 ± 15.4 | 15.2 ± 18.0 | 13.8 ± 16.8 |
| *p-value* (tapentadol vs. methadone) | 0.36 | 0.39 | 0.45 | 0.83 | 0.84 |
| *p-value* (tapentadol vs. hydromorphone) | 0.87 | 0.83 | 0.78 | 0.56 | 0.41 |
| *p-value* (tapentadol vs. oxycodone) | 0.87 | 0.85 | 0.80 | 0.52 | 0.45 |
| *p-value* (tapentadol vs. fentanyl) | 0.84 | 0.78 | 0.79 | 0.61 | 0.52 |
| *p-value* (methadone vs. hydromorphone) | 0.91 | 0.96 | 0.99 | 0.99 | 0.94 |
| *p-value* (methadone vs. oxycodone) | 0.97 | 0.98 | 0.99 | 0.97 | 0.94 |
| *p-value* (methadone vs. fentanyl) | 0.95 | 0.98 | 0.99 | 0.99 | 0.97 |
| Patients without numbness |  |  |  |  |  |
| Tapentadol (n = 36), mean ± SD | 17.5 ± 15.7 | 5.0 ± 10.0 | 5.0 ± 10.0 | 5.0 ± 10.0 | 2.5 ± 5.0 |
| Methadone (n = 37), mean ± SD | 25.6 ± 28.7 | 11.8 ± 17.1 | 10.3 ± 12.8 | 6.4 ± 8.8 | 3.6 ± 6.6 |
| Hydromorphone (n = 40), mean ± SD | 17.5 ± 7.2 | 7.5 ± 10.1 | 6.1 ± 8.6 | 4.6 ± 8.7 | 3.6 ± 6.3 |
| Oxycodone (n = 35), mean ± SD | 17.8 ± 21.8 | 7.0 ± 15.6 | 6.6 ± 15.8 | 5.2 ± 10.4 | 3.3 ± 5.5 |
| Fentanyl (n = 33), mean ± SD | 17.0 ± 5.4 | 7.5 ± 7.5 | 7.5 ± 7.5 | 5.3 ± 7.3 | 3.8 ± 6.5 |
| *p-value* (tapentadol vs. methadone) | 0.71 | 0.78 | 0.84 | 0.99 | 0.99 |
| *p-value* (tapentadol vs. hydromorphone) | 0.99 | 0.99 | 0.99 | 0.99 | 0.99 |
| *p-value* (tapentadol vs. oxycodone) | 0.99 | 0.99 | 0.99 | 0.99 | 0.99 |
| *p-value* (tapentadol vs. fentanyl) | 0.99 | 0.99 | 0.99 | 0.99 | 0.99 |
| *p-value* (methadone vs. hydromorphone) | 0.70 | 0.97 | 0.96 | 0.99 | 0.99 |
| *p-value* (methadone vs. oxycodone) | 0.69 | 0.95 | 0.97 | 0.99 | 0.99 |
| *p-value* (methadone vs. fentanyl) | 0.80 | 0.98 | 0.99 | 0.99 | 0.99 |
| Dunnett's test.  Abbreviations: SD, standard deviation; vs., versus. | | | | | |
